# Microsporidiosis in Vertebrate Companion Exotic Animals

**DOI:** 10.3390/jof2010003

**Published:** 2015-12-24

**Authors:** Claire Vergneau-Grosset, Sylvain Larrat

**Affiliations:** 1 Zoological medicine service, Faculté de médecine vétérinaire, Université de Montréal, 3200 Sicotte, Saint-Hyacinthe, QC J2S2M2, Canada; 2 Clinique Vétérinaire Benjamin Franklin, 38 rue du Danemark, ZA Porte Océane, 56400 Brech, France; sylvainlarrat@yahoo.fr

**Keywords:** microsporidia, *Encephalitozoon*, *Pleistophora*, albendazole, fenbendazole

## Abstract

Veterinarians caring for companion animals may encounter microsporidia in various host species, and diagnosis and treatment of these fungal organisms can be particularly challenging. Fourteen microsporidial species have been reported to infect humans and some of them are zoonotic; however, to date, direct zoonotic transmission is difficult to document *versus* transit through the digestive tract. In this context, summarizing information available about microsporidiosis of companion exotic animals is relevant due to the proximity of these animals to their owners. Diagnostic modalities and therapeutic challenges are reviewed by taxa. Further studies are needed to better assess risks associated with animal microsporidia for immunosuppressed owners and to improve detection and treatment of infected companion animals.

## 1. Introduction

Microsporidia are eukaryotic organisms with the smallest known genome [1]. Microsporidia had been classified as amitochondriate due to their lack of visible mitochondria, but sequences homologous to genes coding for mitochondria have since been discovered in their genome and remnants of mitochondria have been visualized in their cytoplasm [2]; therefore, they have been reclassified as fungi based on phylogenic analysis of multiple proteins in their genome, clustering preferentially with fungal proteins [2,3]. Currently, microsporidia are considered atypical fungi without mitochrondria [2,4,5,6]. More than 1200 species are described, some of which are able to infect multiple host species [7,8]. A comparative review is relevant for microsporidia, as diagnostic tests and potential treatments are common to many hosts, and veterinarians caring for companion animals may encounter these fungi in various host species.

Microsporidia were first reported in the middle of the 19th century when an outbreak threatened the silkworm industry [9,10]. The majority of microsporidia species infect arthropods [11]. Most species infecting vertebrate hosts are found in fish [11]; however, companion animals that are commonly seen by exotic veterinary practitioners, such as rabbits, rodents, birds and reptiles, can also be infected by microsporidia. Most microsporidia are obligate intracellular fungi [8,12]. Members of this phylum exclusively reproduce asexually [13]. Some microsporidia form xenomas, which are single hypertrophied host cells containing multiple microsporidia. Xenomas can reach 14 mm in diameter [14], making them macroscopically visible. These large xenomas have been described as “pseudotumors” [8,12]. Microsporidial spores are smaller than their protozoan counterparts and differ from them by being Gram-positive or Gram-variable [12]. Microsporidia often represent a diagnostic challenge because many microsporidial genera do not form xenoma and infection may be subclinical.

A typical microsporidian cycle involves three phases: the infective phase, which is characterized by polar tube extrusion and injection of the sporoplasm into the cytoplasm of a host cell; the proliferation phase or merogony; and, finally, the spore-forming phase or sporogony [12,15]. Spores can survive for over a year in the environment [16] and disinfection is necessary to inactivate them. Ultraviolet-sterilization of water at 6 mJ/cm^2^ has been shown to eliminate some microsporidial spores of concern to human health [16]. In contrast, some microsporidia are highly resistant to chlorine disinfection [17]. Spores measure 3 to 5 μm in diameter and are difficult to identify by light microscopy. Microsporidian genera can be classified based upon their ability to form xenomas [8] and upon whether or not spores develop in membrane-bound packets called sporophorous vesicles [12]. Identification of the precise species of microsporidia is based on the target tissue infected, the size and morphology of the spores, including the number of nuclei [18], the presence or absence of sporophorous vesicles and diplokarya or paired nuclei [14], the number of spores in sporophorous vesicles, if present [13], and the structure of xenomas, if present [14]. Currently, molecular diagnostic tests using polymerase chain reaction (PCR) with primers targeting the small subunit ribosomal RNA gene are required to confirm identification [19].

Diagnosis can be reached via direct visualization of typical spores by light or electronic microscopy, via cell culture, tissue histology, immunohistochemistry, or via serologic [20] and molecular techniques [21]. Microsporidian spores are Gram-positive or Gram-variable [14], birefringent and some of them are acid-fast-positive [12]. Chitin-binding fluorochromes [12,22,23], chromotrope stains [21], modified trichrome stain, and Giemsa stain [21] can also be used to stain microsporidial spores on smears and stool samples, which has been demonstrated in humans [22]. Notably, fluorescent stains, such as the Luna calcofluor stain, can be used since microsporidian spores contain chitin, but these stains require immunofluorescence microscopy and may lead to false positives since other fungal spores also fluoresce with these stains [24]. Molecular diagnostic techniques include PCR of the small subunit rRNA gene using PMP1 and PMP2 primers [25,26]. It is challenging to determine the actual prevalence of microsporidia infection in a population as shedding is typically intermittent [24] and transit of the fungi through the feces cannot be differentiated from digestive infection based on fecal detection. Of note, the intensity of fecal shedding, and thus the sensibility of fecal screening, has been correlated with severity of infection by intestinal microsporida in humans but has not been correlated with the occurrence of diarrhea [27]. These challenges are illustrated by the high variability of prevalence rates, even from studies derived from the same geographic area [28].

Fourteen microsporidial species have been shown to infect humans [28,29], with 90% of human cases being caused by *Enterocytozoon bieneusi* [30]. Suspected risk factors for microsporidiosis in human include young age [31,32], immunosuppression (e.g., acquired immune deficiency syndrome with low CD4 T lymphocytes [33]), treatment with immunosuppressive drugs [31], especially after organ transplantation [34], wearing contact lenses [35], and exposure to contaminated environments [36]. Among HIV-positive patients, reported risk factors also include poor sanitation [33], living in a rural area [33], consumption of watermelon [33], use of injectable drugs [37], exposure to contaminated water in hot tubs and spas, and occupational contact with water [37]; some of these factors are still debated. More information is needed regarding risk factors of developing microsporidiosis in animals, but immunomodulation by cyclophosphamide [38] or dexamethasone [39] has been shown to be a risk factor in rabbits.

A recent study involving more than 200 patients investigated fecal detection of microsporidia in healthy humans and immunocompetent patients presenting with diarrhea: interestingly, the prevalence of microsporidia was higher in the healthy group with 45% of healthy patients shedding microsporidia [24]. This suggests that digestive microsporidia are often asymptomatic in humans [20]. Among HIV-positive patients, infection with microsporidia did not significantly shorten survival in a case-control study [33].

The zoonotic potential of microsporidia was first suggested in 1995 [40]. However, the clinical importance of this potential risk remains to be elucidated, as there is no formal proof of zoonotic transmission of microsporidia [27]. A zoonotic disease requires: (1) infection of an animal by the microorganism, with or without disease; (2) transmission of the microorganism from the animal to a human, and (3) infection of a human and development of a disease [41]. Numerous reports show that human-infecting microsporida can be detected in the feces of animals [42,43,44,45]. However, demonstrating infection of animals requires tissue histopathology and confirmation of a human-infecting strain of microsporidia in the tissue by molecular techniques; microsporidia can indeed travel through the digestive tract of animals without an actual host infection. In this case, animals do not amplify microsporidia. It would, therefore, be misleading to claim a zoonotic risk; in fact, the risk of human infection may be the same in the presence of animal as it is through environmental exposure. A nested cohort study conducted in HIV-positive patients in Peru showed a statistical association between infection by *Enterocytozoon bieneusi* genotype 1 and contact with rabbits, ducks, sheep and some domestic animals [33]. However, the same study also highlighted a statistical association between poor sanitation at home and microsporidiosis [33]. This could be an important confounding factor as the limited availability of running water and contact with farm animals are both more likely to occur in rural areas [33]. In addition, the authors of the papers acknowledged that *E. bieneusi* genotype 1 infection has not been reported in animals [33]. Another genotype of *E. bieneusi*, named Peru 16, has been isolated from a diarrheic child and seven subclinical guinea pigs of the same household [46]. Owing to the growing interest about zoonotic microsporidia and to their pathogenicity in veterinary species, veterinarians should be familiar with the challenges associated with microsporidial infection diagnosis and treatment in companion animals. This review summarizes the last advances in the diagnosis and treatment of companion exotic animal microsporidiosis.

## 2. Ornamental Fish Microsporidiosis

### 2.1. Agents and Disease in Fish

More than 14 genera of microsporidia infect freshwater and marine fish [8], teleosts, and elasmobranchs [47]. Cases in elasmobranchs are very uncommon [48]: microsporidial infection in the free-ranging common stingray (*Dasyatis pastinaca*) in Turkey has been reported to cause nodules around the disc [49], while a systemic and fatal infection has been reported in the captive leopard shark [48]. Reported microsporidian genera in fish include *Dasyatispora*, *Glugea*, *Heterosporis*, *Ichthyosporidium*, *Kabatana*, *Loma*, *Nucleospora*, *Pleistophora*, *Spraguea* [1], *Tetramicra* [45], and *Pseudoloma* genera, among others [12]. Most genera are specific to a fish species [8], while genera that are able to form xenomas, such as *Glugea*, can infect a broad range of fish species [50]. Zoonotic *Pleistophora* spp. have been suspected in fishermen [51].

Microsporidiosis has been reported in captive and wild fish [52]. In ornamental fish, microsporidiosis is particularly common in zebrafish (*Danio rerio*) infected by *Pseudoloma neurophilia* [53] and *Pleistophora* spp. [54] and in tetra infected with *Pleistophora hyphessobryconis*, which causes the disease known as “neon tetra disease” [12]. Infection by *P. neurophila* has been reported in 74% of the laboratory facilities examined through the Zebrafish International Resource Center pathology service in 2010 [55]. Most fish microsporidia have a preferential target site of infection, including striated muscles [49], coelomic organs, gills, the digestive tract, ovaries, and the central nervous system [55].

### 2.2. Diagnosis of Fish Microsporidiosis

For xenoma-forming species, such as *Glugea* spp., external masses can be detected macroscopically. Skin scrapes may facilitate detection of the typical spores in tetra, although xenomas are absent with *Pleistophora* spp. infections [21,22]. Spores can develop within three to four weeks after infection in some microsporidia species [56] and shedding may occur through the feces, urine and sex products during spawning depending on the infected organs [50]. In addition, spores may be released from lesions on the body surfaces ante-mortem or post-mortem [13,48]. Chitin-binding fluorochromes are the most sensitive stains to highlight fish microsporidial spores in histologic sections [22,23]. *In vitro* cell culture of fish microsporidia has been historically more difficult to achieve than for human- or insect-infecting microsporidia [57].

### 2.3. Treatment of Piscine Microsporidiosis

Disinfection of a fish tank or exhibit after an outbreak of microsporidiosis may be challenging [17]. While 5 ppm of chlorine, a typical concentration applied to tap water, is sufficient to inactivate *Encephalitozoon cuniculi* after 10 min, 1500 ppm of chlorine is needed at neutral pH to achieve more than 95% *Glugea* *anomala* spore inactivation, and higher pH decreases the efficacy of this disinfectant [17]. Ultraviolet sterilizers used in aquarium life support systems are an important means of preventing the spread of microsporidiosis in a facility through water sterilization [55,58]. In addition, surface disinfection techniques applied to fish eggs, such as iodine treatments in salmon and sodium hypochlorite bath treatments in zebrafish, have been unsuccessful to date [54]. This is expected because some microsporidia are thought to be transmitted vertically and can be present within the eggs [54].

Microsporidia may also be transmitted orally. Prevention of infection in piscivorous captive fish may require inactivating food-borne microsporidial spores. Inactivation of spores of *Spraguea lophii* in anglerfish can be achieved by freezing at −20 °C for 48 h, treatment at 60 °C for 15 min and microwaving at 750 watts for more than 60 s [59].

Treatment of infected fish has only been partially effective. Albendazole and fumagillin have been shown to decrease both the number of xenoma and the proportion of fish with xenomas in a school of rainbow trout (*Oncorhynchus mykiss*) infected by *Loma salmonae*, contrary to pyrimethamine, sulphaquinoxaline, amprolium and metronidazole [60], which have all been shown to be ineffective in treating active infections. However, fumagillin has also shown severe adverse effects in rainbow trout at efficacious dosages [61]. Fumagillin was initially used for the treatment of bee nosemosis [62]. It is a molecule produced by *Aspergillus fumigatus* [18]. Fumagillin is not approved for human or veterinary use and should not be used for food-animal species including fish [61]. In humans, fumagillin has been shown to cause reversible thrombocytopenia [18]. Quinine hydrochloride and decreasing the water temperature can delay the formation of xenomas in fish [63]. In addition, monensin has been shown to prevent infection by *L. salmonae* [64]. Although approved for some food-animal species [65], monensin is not approved for use in fish destined for human consumption in the United States [65], Europe [66] and Canada [67].

Experimental vaccination trials of salmonidae against microsporidial gill disease with spore-based inactivated or low-virulence live *Loma salmonae* vaccines have shown promising results [68,69] but these vaccines are not currently commercialized.

## 3. Rabbit and Rodents Encephalitozoonosis

### 3.1. Agent and Disease in Rabbits and Rodents

*Encephalitozoon cuniculi* is the most commonly reported microsporidia in non-human mammals [18]. To date, three strains of *E. cuniculi* have been officially described based on the number of repeated elements 5′-GTTT-3′ in the internal transcribed spacer (ITS) [7,40]. Strain I is typically found in rabbits, strain II has been described in rodents, and strain III has been reported in dogs and cotton-top tamarins [7,70,71]. Strains I and III have been shown to cause infection in humans [7,72], while strain II has also been detected in the stools of an HIV-infected patient [73]. In addition, novel strains have been reported in a human patient with a renal transplant [74], in cats [75], and in bearded dragons [76]. *E. cuniculi* is transmitted either vertically or horizontally in rabbits [7], including transmission via ingestion or inhalation of spores excreted in the urine of infected rabbits [77]. Spores can survive outside of the host for six weeks at 22 °C and less than a week at 4 °C [77]. Other microsporidia, such as *E. bieneusi*, have also been isolated from the feces of healthy rodents [46].

Most infections by *E. cuniculi* are subclinical [77] and some studies have reported seroprevalence ranging from 45% [78] to 89% in healthy rabbits [79]. T-cell immunity is thought to play a critical role in disease manifestation in rabbits and rodents, as in humans [80]. Alternatively, infection of target organs including the central nervous system, lungs and kidneys in rabbits and, less commonly, guinea pigs [77] can lead to cell rupture, inflammation, and clinical signs. Neurological signs are commonly reported and have been observed in some animals following experimental infection [81]. Interestingly, although vestibular disease is reported as a common neurological sign in rabbits infected with *E. cuniculi* [82], the lesions seem to affect the neocortex more often than the brainstem where the vestibular nuclei are located [83]. More precise histologic evaluation of the vestibular nuclei as well as systematic evaluation of the ear would be warranted to refine the post-mortem diagnosis of vestibular disease etiology, since the presence of *E. cuniculi* in the central nervous system may be incidental. Renal signs are also suspected but one study failed to demonstrate proteinuria in seropositive rabbits [84]. In mice, typical lesions include granulomatous hepatitis, interstitial nephritis, and meningoencephalitis. In immunocompromised mice, ascites is a common clinical sign of encephalitozoonosis [84]. Lens infection likely results from vertical transmission and can ultimately lead to uni- or bilateral phacoclastic uveitis [15,77,78,85,86]. Abortion and neonatal death have also been associated with *E. cuniculi* [77].

### 3.2. Diagnostic Challenge in Rabbits

Diagnosing encephalitozoonosis in rabbits is particularly challenging, due to a short excretion period and because neither histopathologic findings [87] nor serologic titers [81] correlate well with the severity of clinical signs. Since encephalitozoonosis is considered one of the most common causes of neurologic clinical signs in companion rabbits [82], veterinarians should be aware of these diagnostic challenges.

#### 3.2.1. Antibody Detection, Electrophoresis and Acute Phase Proteins

Available serologic tests for detection of antibodies against *E. cuniculi* in rabbits include carbon immunoassay [81,88], enzyme-linked immunoassay (ELISA) [80], and indirect immunofluorescent assay [89,90]. Antibodies become detectable two to four weeks after infection [81], which is up to two weeks before spores are detected in urine and tissues [91]. Maximal titers are detected six to nine weeks after infection and immunoglobulin M (IgM) decreases to 0 by 35 days post-infection [87]. Immunoglobulin G (IgG) becomes the dominant immunoglobulin around 17 days post-infection and can continue to be produced during chronic infection [87]. Sustained antibody titer has been reported to last more than seven years after experimental subcutaneous administration of inactivated spores [90]. Conversely, short antibody responses have also been documented [81]. Of note, offspring from seropositive dams may display detectable colostral antibodies for four weeks before titer becomes undetectable.

*E. cuniculi*-negative rabbit colonies have been established for research purposes, but many companion rabbits are seropositive to *E. cuniculi* [7], with a seroprevalence ranging from 29.5% to 73% depending on the study area and on the serologic assay used [78,87,92,93,94]. Seropositivity is very frequent in apparently healthy rabbits [95]. Some studies report significantly higher seroprevalence rates in symptomatic rabbit populations than in subclinical rabbits [78,93], whereas no statistically significant difference in seroprevalence between healthy and symptomatic rabbits was present in one study [95]. However, serologic titers correlated strongly with lesion intensity in the same study [95].

A panel including an ELISA serologic test associated with the acute phase proteins C-reactive protein is offered by the University of Miami laboratory [89,96,97]. This panel has been evaluated based on clinical suspicion of encephalitozoonosis in rabbits, rather than actual confirmation of infection [89,96,97]. Titers above the cut-off for IgM have been found in 24% of clinically normal rabbits [89]. It is expected that some rabbits previously infected by *E. cuniculi* could be seropositive and have concomitantly high C-reactive proteins due to their current disease and the reported specificity of 100% is therefore surprising in these cases [97]. A study has shown a 10-fold mean increase of the C-reactive protein concentration in rabbits suspected to be infected by *E. cuniculi*, based on the description of those clinical signs which were not explained by another causative agent [89]. Histologic diagnosis was available for only three rabbits suspected to be infected by *E. cuniculi* in this study [89]. Due to the study design, it is unknown whether the clinical signs of these rabbits were actually associated with *E. cuniculi* infection. Conversely, haptoglobin and serum amyloid A were not increased in the rabbit suspect group [89]. The significance of seropositivity at the individual level is controversial in rabbits. Experimental infection of rabbits has shown that antibody titer intensity is not correlated with recovery of the organism from brain tissue [81]. A study comparing companion rabbits suspected to be infected by *E. cuniculi* with non-suspect rabbits has shown that suspect rabbits had antibody titers 1.7 times higher than rabbits from the non-suspect group with a mean titer of 1:1324 in the suspect group [96]. However, only 79% of the rabbits included in the “suspect” group were seropositive, which could be due to a lack of seroconversion of some infected rabbits or to misclassification of some rabbits that were not, in fact, infected by *E. cuniculi* [96]. In addition, the inter-assay coefficient of variation of the serologic test was not provided, and this difference is usually below one dilution and could therefore represent inherent test variation. In another study evaluating the same serologic panel, the presence of concomitantly increased IgG, IgM, and C-reactive protein concentrations was reported to have a specificity of 100% in detecting encephalitozoonosis in rabbits [97]. This result, almost too perfect to be trusted, raises some concerns regarding the validity of the study design. It seems logical that this perfect specificity would be an illusion: for example, it is expected that many individuals from a population of naïve juvenile rabbits will display high IgM and IgG concentrations, since *E. cuniculi* is a common fungus in their environment. If they are exposed concomitantly to other pathogens causing neurologic signs in rabbit kits, such as *Pasteurella multocida* causing suppurative encephalitis, C-reactive protein will also increase. They will therefore be presented with the perfect picture of the *E. cuniculi* positive rabbit based upon the serologic panel, yet their true pathologic condition would be pasteurellosis and their therapeutic plan may suffer from this mistaken result. Practically, most veterinarians will formulate a basic treatment plan before receiving serologic test results because of the delay in result reporting. However, veterinarians should be aware of the imperfect specificity and limited sensitivity [97] of this serologic panel. Post-mortem confirmation of infection using PCR was not available to evaluate if inclusion criteria were valid in this study. Overall, more studies are needed to confirm the value of serologic and acute phase protein panels: these studies should include cases with a definitive diagnosis rather than strong anecdotal information, as acknowledged by the authors of the study about the serologic panel [97].

Serum and plasma protein electrophoresis has been suggested as an adjunctive diagnostic test for encephalitozoonosis [96]. Rabbits classified as *E. cuniculi* suspects and other sick rabbits had a significantly lower albumin-to-globulin ratio, total protein, and α2-globulin fractions with a higher γ-globulin fraction compared to clinically normal groups [96]. However, no specific electrophoretic changes enabled differentiation of *E. cuniculi*-suspect rabbits from other sick rabbits [96] and this test was therefore not clinically relevant to orient the diagnosis toward encephalitozoonosis in sick rabbits.

Detection of antibodies against *E. cuniculi* in rabbit urine has been investigated [98]. Immunoglobulin G and fragments of immunoglobulin G against the polar tube and spore wall can be measured separately [98]. In a colony of seropositive rabbits, only some seropositive individuals had detectable urinary immunoglobulins [98]. The authors interpreted this test as being possibly more specific than blood serology to detect renal disease associated with *E. cuniculi*; detection of urinary antibodies may indicate impaired glomerular filtration associated with multiplication of *E. cuniculi* in the kidney and resulting in excretion of antibodies [98]. However, this test requires further validation [98], especially since histologic lesions are more commonly tubular than glomerular with *E. cuniculi* [83].

Cerebrospinal fluid protein evaluation has been compared between 23 laboratory seronegative rabbits and 20 *E. cuniculi*-seropositive and *Toxoplasma*-negative companion rabbits with neurologic clinical signs including 12 with confirmed postmortem infection [99]. Protein concentration and cerebrospinal fluid leukocyte counts were significantly higher in infected rabbits than in healthy rabbits [99]. It would be interesting to evaluate the results of cerebrospinal cytologic evaluation in rabbits affected by other neurologic disease to compare them to those of *E. cuniculi*-infected rabbits. However, this procedure is rarely performed in companion rabbits due to its inherent risks, which limits the applications of this technique.

#### 3.2.2. Antemortem Antigenic Tests

**Figure 1:**
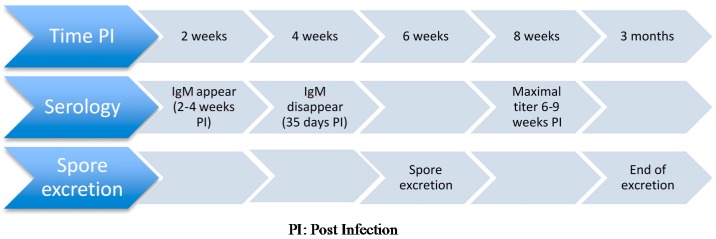
Sequence of events used for diagnosis of *Encephalitozoon cuniculi* in an immunocompetent companion rabbits.

Antemortem antigenic tests include detection of *E. cuniculi* spores in the urine via real-time PCR. The main limitation of this technique is the short temporal period of spore excretion in rabbits, which limits the test sensitivity. Seroconversion precedes renal shedding and is still detectable afterwards [77] (Figure 1). Whether or not shedding increases in immunocompromised rabbits is debatable: some authors have not shown any effect of dexamethasone administration on spore excretions [39] while others suggest the contrary [100], as dexamethasone has been reported to increase spore excretion in mice [101,102]. Urinary spore excretion typically occurs between four weeks and 12 weeks post-infection [77], which may be earlier than the development of clinical signs.

In the case of phacoclastic uveitis, detection of *E. cuniculi* spores in the lenses of rabbits is also performed by conventional PCR or immunohistochemistry after enucleation or lens phacoemulsification [78,103]. In a study performed in companion rabbits suspected to be infected by *E. cuniculi*, a PCR test successfully detected *E. cuniculi* in the lenses of four out of five patients while 32 urine samples and 12 cerebrospinal fluid samples were negative [78].

#### 3.2.3. PostMortem Diagnosis

*E. cuniculi* can also be challenging to diagnose postmortem [104]. The use of histopathology as the sole means of spore detection is at once insensitive and non-specific [83]. Histologic lesions may include nonsuppurative granulomatous encephalitis [15], spinal root inflammation, chronic lymphoplasmocytic to granulomatous interstitial nephritis [82], pneumonitis, myocarditis, hepatitis, splenitis, and uveitis [77]. Renal lesions have been detected as early as one month after experimental infection while cerebral lesions are thought to develop later on, after about two to three months [15,81]. In a study comparing patients’ neurologic signs to their postmortem lesions, no correlation was found between the severity of clinical signs and histopathologic lesions [87]. The Ziehl-Neelsen stain enabled detection of a higher number of spores than the trichrome stain, but the difference in sensitivity between the two staining techniques was not statistically evaluated [87]. Spores also stain with Goodpasture’s carbol fuchsin [77].

The proposed gold standard for diagnosing *E. cuniculi* infection is real-time PCR on tissue samples, which is currently the best technique of diagnosis. One study compared immunohistochemistry (IHC), real-time PCR, and histopathology of kidney, brain and lung tissue [83]. Histopathology was found to be less sensitive than immunohistochemistry, which was less sensitive than real-time PCR [83]. Brain and kidney were the most sensitive tissues for *E. cuniculi* detection. Within the brain, lesions were most commonly localized in the gray matter of the neocortex [83]. Within the kidney, acute lesions were mainly localized in the medulla while chronic lesions were mostly localized in the renal cortex [83]. In another study comparing the sensitivity of IHC and *in-situ* hybridization in infected rabbit tissue with electron microscopy, confirmation IHC was shown to be more sensitive than *in-situ* hybridization [104]. However IHC sensitivity was only 37% on kidney and brain samples and 60% on lens samples in this study, which included naturally infected companion rabbits [104].

### 3.3. Therapeutic Challenges

Due to the challenges associated with *E. cuniculi* diagnosis, especially antemortem, evaluating treatment efficacy is particularly difficult. Similar to diagnostic test evaluation, many authors have attempted studies in rabbits based on a presumptive infected status.

Proposed treatments for encephalitozoonosis in rabbits include benzimidazoles such as fenbendazole [105], oxibendazole, and albendazole [106]. Some fluoroquinolones have been suggested to have an inhibitory effect *in vitro* and *in vivo* in mice against *Encephalitozoon intestinalis* and some other microsporidia [107]. However, it should be noted that ciprofloxacin, a metabolite of enrofloxacin, did not have a significant effect when compared to norfloxacin and ofloxacin. The effect of fluoroquinolones on *E. cuniculi* has not been investigated to the authors’ knowledge and *in vivo* studies in rabbits using dosages previously established by pharmacokinetic studies [108] would be needed to investigate the effect of ofloxacin in this circumstance.

Fenbendazole has been shown to have a prophylactic and therapeutic effect against *E. cuniculi* in rabbits [105]. Fenbendazole metabolism has been investigated in rabbits [109,110]. Fenbendazole has been suggested as a superior treatment option compared to oxytetracycline in rabbits naturally infected by *E. cuniculi* based on survival rate and clinical improvement of their neurological score at 10 days [100]. However, postmortem diagnosis in this study was based exclusively on histopathology; therefore, some rabbits in this study could have been infected with other agents responsible for causing multifocal granulomatous encephalitis [100]. In addition, this study did not include a control group and the treatment attribution was neither randomized nor double-blinded for fenbendazole [100]. Although previously thought to be innocuous [105], albendazole, fenbendazole, and oxibendazole have all been reported to cause aplastic anemia and pancytopenia in rabbits [111]. In a case series of 13 suspected cases of benzimidazole intoxication, only one rabbit survived after receiving intensive supportive care including blood transfusions and erythropoietin administration [111]. Therefore, complete blood count rechecks or serial hematocrits are recommended as part of the follow up to benzimidazole treatment in rabbits. Albendazole should not be used in pregnant rabbits as it has been reported to be teratogenic and embryotoxic [112]. In addition, treatment with albendazole failed to clear microsporidia from experimentally infected rodents and further immunosuppression resulted in reactivation of *E. cuniculi* in an experimental model [113], showing the ineffectiveness of this approach [113].

Clinically, the use of corticosteroids in the treatment of encephalitozoonosis in rabbits seems contraindicated, as rabbits are particularly susceptible to the immunosuppressive effects of corticosteroids, which could amplify the parasitic infection [39]. In addition, a prospective study in a rabbit cohort did not show any impact of dexamethasone on clinical signs, as dexamethasone neither increased rabbit survival rate nor prolonged duration of survival [100]. Again, this study did not thoroughly confirm infection by *E. cuniculi* postmortem as no molecular testing was attempted on tissue and central nervous system lesions consistent with encephalitozoonosis were attributed to *E. cuniculi* even in seronegative rabbits [100]: hence, the treated group could have included rabbits with toxoplasmosis, among others. It is unknown whether the addition of dexamethasone can decrease albendazole elimination rate in rabbits as observed in humans [114,115]. Currently, dexamethasone is not recommended as an adjunctive treatment of encephalitozoonosis in rabbits.

Future therapeutic tools for use in rabbits may include drugs that have been tested in rodents and fish. New drugs under investigation include p38 mitogen-activated protein kinase (MAPK) inhibitors [116]. This class of drugs has been shown to prevent clinical signs in mice experimentally infected two days prior to administration of these drugs [116]. This class of drug is currently experimental and cannot be used clinically by veterinarians. Fumagillin-related compounds have been shown to inhibit *E. cuniculi in vitro* [106,117], but are not used currently in rabbits to the authors’ knowledge, although they have been suggested [112].

Many drugs have been cited for potential treatment of rabbit encephalitozoonosis but should not be used. Although used to treat microsporidiosis in fish and anecdotally cited in rabbits, toltrazuril and its metabolite ponazuril have not been tested *in vivo* in rabbits for the treatment of encephalitozoonosis [118]. *In vitro*, toltrazuril and ronidazole did not show any effect against *E. cuniculi* [106]. Sulfonamides and paromomycin have not shown *in vitro* efficacy against *E. cuniculi* [119], although they are occasionally cited as treatment options [100]. Fluoroquinolones have been suggested as a treatment for human microsporidiosis but applications in rabbits have not been investigated [107]. Tetracycline derivates have been shown to be only partially effective and clinical experience does not support their use in rabbits [100].

Regarding environmental disinfection, *E. cuniculi* has been shown to be susceptible to exposure for 10 minutes to common disinfectants [120,121], including bleach 0.1%–10%, quaternary ammonia, 70% alcohol [121], phenolic derivates, sodium hydroxide, chloramine, iodophores, hydrogen peroxide, and amphoteric surfactants [112].

Experimental vaccination of New Zealand White rabbits with inactivated *Encephalitozoon* spp. spores induced a seroconversion and antibodies were predicted to last for about seven years [90]. However, efficacy of this vaccine against microsporidial infection has not been tested as the main goal of the study was to develop a better source of anti-microsporidial serum for immunocompromised persons [90].

## 4. Avian, Reptile and Amphibian Microsporidiosis

### 4.1. Microsporidia in Amphibians

Microsporidia have been uncommonly reported in amphibians [122] both in anura [123,124,125] and urodela [126]. *Pleistophora* spp. has been associated with myositis in toads [123] and with kyphosis and epaxial myositis in San Marcos salamanders (*Eurycea nana*) [126]. Digestive xenomas have been described in *Bufo marinus* tadpoles infected with *Alloglugea* sp. [124]. Necrotizing hepatitis was described with unidentified microsporidia in tadpoles of the endangered mountain yellow-legged frogs (*Rana muscosa*) [127] and intestinal xenoma have been reported in subclinical cane toad tadpoles (*Rhinella marina*) [124]. In the cases of digestive lesions, typical spores may be detected on fecal examination [122]. Spores may also be visualized on blood smear in case of septicemia [115]. Diagnosis can be made via histology with visualization of Gram-positive spores in tissues, electron microscopy, and molecular techniques including PCR followed by sequencing [128]. Chloramphenicol sodium succinate and topical oxytetracycline hydrochloride, and polymyxin B sulfate have been suspected to inhibit microsporidial spore production in amphibians in a single case series; however, supportive care and other treatments were also administered in these American giant tree frogs and the report did not include a control group [125].

### 4.2. Microsporidia in Reptiles

Microsporidia are rarely reported in squamates and rhynchocephalia [8,16,76,128,129] but have not been reported in chelonians to the authors’ knowledge. Some microsporidia species have only been described in reptiles, such as *Glugea danilewskyi* [130], *Pleistophora atretii* [131] or *Encephalitozoon lacertae* [132], while others, such as *Heterosporis anguillarum*, are suspected to be transmissible between fish and reptiles [8]. A strain of *E. cuniculi* distinct from mammalian *E. cuniculi* has been described in bearded dragons (*Pogona vitticeps*) [76] but it is unknown whether this species was the same as those in earlier reports as molecular techniques were not available [129]. Similarly, reported *Encephalitozoon* sp. in African skinks (*Mabuya perrotetii*) [133] could be the same species as microsporidia reported in *Podarcis muralis* based on morphology [132]. Transmission of microsporidia in reptiles has been poorly studied and infection through ingestion of infected prey items and vertical transmission are suspected [13].

Systemic infections have been reported in bearded dragons with non-specific clinical signs including lethargy, anorexia, and severe polydipsia [76,129]. Granulomatous lesions were detected in the kidneys, liver, spleen, lungs, adrenal, gonads, fat bodies, and digestive tract mucosa [76]. Some microsporidia have also been found in endothelial cells of the central nervous system capillaries [129]. Unlike bearded dragons, a localized form of microsporidiosis characterized by myositis and thickening of the coelomic wall cranial to the cloaca have been reported in a gartersnake (*Thamnophis sirtalis*) [8] and in tuataras (*Sphenodon* *punctatus*) [128]. Interestingly, while microsporidia are thought to be intracellular fungi, some extracellular forms of *Heterosporis anguillarum* were noted in the gartersnake [8]. Microsporidia have been detected in the feces of 19 subclinical snakes and a subclinical lizard from a zoological collection, which died of other causes; this finding has led to the suspicion that microsporidia could be opportunistic pathogens in squamates [16]. Alternatively, the spores observed could originate from the reptiles’ food and represent a passage through the digestive tract rather than a true infection.

Most cases have been described postmortem in reptiles and, to the authors’ knowledge, no treatment attempt has been reported to date. Diagnostic tests include histopathology, electron microscopy, and molecular techniques as described in other vertebrates [8,76,129].

### 4.3. Microsporidia in Birds

#### 4.3.1. Agents and Disease in Birds

Microsporidia carried by birds have been studied to evaluate zoonotic risks associated with wild [134,135] and captive birds [136,137,138,139]. When investigating feral urban pigeons and exotic birds seized from illegal trade in Brazil, shedding prevalence was found to be higher in pigeons, with 31% of pigeon stools being positive for microsporidia, although some birds from this study could have been sampled repeatedly [140]. Similarly, 29% of 124 individually sampled pigeons in Spain were found to shed microsporidia [141]. However, many studies have shown only excretion of microsporidial spores in bird stools, which could represent a simple transit through the digestive tract rather than an actual infection as demonstrated in naturally exposed budgerigars [142]. Hence, the detection of the most common microsporidia isolated from humans, *E. bieneusi*, in birds living in close contact with people, such as urban pigeons [141] and domestic chickens [143], may in fact reflect a transit rather than a true zoonotic risk. The distinction between mechanical vector and infected bird is especially important when very sensitive molecular screening tools such as PCR are being used [141].

Actual infection of birds associated with disease and pathologic changes has been confirmed in lovebirds infected by *Encephalitozoon* sp. [144,145,146,147], budgerigars [148], eclectus parrots [149], double-yellow headed Amazon parrots [150,151], an umbrella cockatoo [62], a yellow-streaked lory [152], an ostrich [25,153], and Anna’s hummingbirds [26] infected by *Encephalitozoon hellem* (Table 1). *E. hellem* is often implicated in human microsporidiosis but, so far, only genotype 1 has been found both in humans and birds, while genotypes 2, 3, and an unnamed genotype have been isolated in only one host group [18]. However, in cases reported before molecular diagnostic tests became available, the genus of microsporidia was assigned based exclusively upon electron microscopy and could be controversial [150]. Fatalities have been associated with microsporidial infections in birds [144,148]. Described clinical signs include diarrhea, hyporexia, respiratory distress [150], and keratoconjunctivitis [62,151]. Differential diagnoses for microsporidiosis should include psittacosis in Psittacidae. In gyrfalcon (*Falco rusticolus*), *E. cuniculi* genotype II has been suspected to cause clinical signs but causality was not demonstrated [154]. Many authors consider avian species to be a possible source of zoonotic microsporidiosis, either by acting as mechanical vectors or by replicating microsporidia [140,151,152,155]. However, mouse inoculation with microsporidia originating from birds has not resulted in infection [144] and no confirmed zoonotic cases have been reported to date.

**Table 1:** Avian species with reported microsporidial lesions and characterization technique used to determine the microsporidial agent.

Avian Host Species	Host Latin Name	Microsporidia Species	Characterization Technique Used	Reference
Anna’s hummingbird	*Calypte anna*	*Encephalitozoon hellem*	PCR	[26]
Budgerigars	*Melopsittacus undulatus*	*Encephalitozoon hellem* and *Encephalitozoon cuniculi*	PCR	[142,148]
Double yellow-headed amazon	*Amazona ochrocephala orathrix*	*Encephalitozoon* sp.	TEM (morphologic)	[150]
Eclectus parrots	*Eclectus roratus*	*Encephalitozoon hellem*	PCR	[149]
Ostrich	*Struthio camelus*	*Encephalitozoon hellem*	PCR	[25]
Peach-faced lovebird	*Agapornis roseicollis*	*Encephalitozoon* sp.	TEM (morphologic)	[145,147]
Umbrella cockatoo	*Cacatua alba*	*Encephalitozoon hellem*	PCR	[62]
Yellow-streaked lori	*Chalcopsitta scintillata*	*Encephalitozoon hellem*	PCR	[152]

#### 4.3.2. Diagnosis of Avian Microsporidiosis

As for other species, diagnostic tests include immunofluorescence and classical stains [26,152] on tissues or body fluids including feces [152], electron microscopy [25,26,154], and molecular tests. In budgerigars, PCR has shown a higher sensitivity for detection in feces than microscopic examination [154]. In addition, the same study demonstrated that PCR was more sensitive than histology and tissue electron microscopy [154]. Fecal shedding has been shown to be intermittent and repeated testing may be required for detection [154]. In a case report, microsporidia were detected on fecal samples via Gram stain but not via PCR, although this was possibly due to intermittent shedding [151]. In this case report, a serologic test developed for rodents to screen for *E. cuniculi* yielded a positive result in an Amazon parrot [151]. No control was available and this test would need to be validated for use in avian species.

#### 4.3.3. Treatment of Avian Microsporidiosis

An umbrella cockatoo has been treated with oral albendazole 25 mg/kg q24h for 90 days and topical neomycin, polymyxin, and bacitracin ointment for keratoconjunctivitis associated with microsporidia [62]. The clinical signs resolved, but three months after the end of the treatment, the contralateral eye developed keratoconjunctivitis. Albendazole treatment was resumed at the same dose for four months and the bird remained free of clinical signs for the following two years [62]. In another case of keratoconjunctivitis in a Psittacidae, oral albendazole at 50 mg/kg q24h for three months, oral itraconazole 10 mg/kg q24h, and fumagillin topical administration every 2 h during the day successfully resolved clinical signs, and post-treatment corneal swab and liver biopsy were negative via PCR and special stains [151]. No adverse effects from albendazole were reported in these two patients.

Of note, fenbendazole, albendazole and other benzimidazoles are toxic in some avian species [156] and can cause medullary aplasia, digestive signs, and death. Toxicity has been reported in some columbiformes [157,158], some vultures [159], storks [159,160], pink-backed pelicans (*Pelecanus rufescens*) [161], a leukemic great-horned owl (*Bubo virginianus*) [162], and, anecdotally, in cockatiels (*Nymphicus hollandicus*) [156] and keas (*Nestor notabilis*) [151]. Because columbiformes are suspected to excrete microsporidia more frequently than other birds [142], finding a viable treatment for this order would be important.

## 5. Conclusions

Microsporidiosis is a rare disease in most companion exotic animals. However, subclinical carriage and shedding have been reported in all vertebrate classes, with or without associated disease. Antemortem diagnosis is complicated by the polymorphism of clinical signs, including masses, myositis, neurologic signs, and any vague clinical signs from systemic disease, which encompasses a wide array of cases seen by veterinarians. Due to the suspected zoonotic potential of some of these fungi, especially in immunocompromised owners, and due to the close contact between companion animals and their owners, clinicians should keep microsporidiosis in differential diagnoses lists. More research would be beneficial to assess treatment efficacy in companion exotic animals.
